# Domain-Adversarial Neural Network for UWB NLOS Identification in Multiple Environments

**DOI:** 10.3390/s26092824

**Published:** 2026-05-01

**Authors:** Suying Jiang, Jiachun Li, Yadong Xu, Yuyang Rong

**Affiliations:** 1 School of Electrical and Control Engineering, Shaanxi University of Science and Technology, Xi’an 710021, China; 250611030@sust.edu.cn (Y.X.); 250612060@sust.edu.cn (Y.R.); 2 College of Big Data and Information Engineering, Guizhou University, Guiyang 550025, China; 3 School of Highway, Chang’an University, Xi’an 710064, China; zhs@chd.edu.cn

**Keywords:** cross-domain, Domain-Adversarial Neural Network (DANN), NLOS identification, Ultra-Wideband (UWB)

## Abstract

Accurate recognition of Line-of-Sight (LOS) and Non-Line-of-Sight (NLOS) signals is crucial for mitigating positioning errors and improving the positioning performance of Ultra-Wideband (UWB) localization systems. Current NLOS identification methods are limited to the specific measurement environments and fail to exhibit effective cross-domain adaptability, being unable to generalize to unseen environments. To address these challenges, we propose a novel NLOS identification strategy based on a Domain-Adversarial Neural Network (DANN). Firstly, aiming at the problem that traditional feature extraction methods fail to capture the deep nonlinear characteristics of Channel Impulse Response (CIR) data, we develop a CNN-DAE-MLP-Attention (CDM) hybrid model for high-quality channel feature extraction, which takes both raw CIR data and handcrafted channel features into account. Secondly, we integrate the CDM model into the DANN framework by replacing its original shallow feature extraction module to further propose the CDMD algorithm; by combining the robust feature representation capability of CDM with the excellent domain adaptation capability of DANN, the proposed CDMD algorithm achieves enhanced performance in cross-domain LOS/NLOS identification. Finally, the effectiveness of the proposed algorithm is verified using measured data from different scenarios. Results demonstrate that the proposed algorithm possesses strong generalization ability. For cross-domain NLOS recognition from underground parking garage to corridor and underground parking garage to lobby, the proposed method achieves accuracies of 77.00% and 72.84%, respectively. Moreover, the results indicate that only a limited number of target-domain samples are sufficient for the model to achieve accurate cross-domain transfer.

## 1. Introduction

With the fast advancement of the Internet of Things (IoT) and intelligent positioning technologies, Ultra-Wideband (UWB) positioning technology has been widely applied in fields such as indoor positioning [1,2,3], industrial navigation, robots, and personnel tracking due to its high temporal resolution, strong resistance to multipath interference, and excellent real-time performance [4,5,6]. However, in complex real-world scenarios, signal propagation paths are easily obstructed by obstacles such as human bodies, walls, industrial equipment, etc., leading to Non-Line-of-Sight (NLOS) propagation [7,8]. NLOS propagation can cause significant ranging errors [9], severely impacting the positioning accuracy of UWB systems. Thus, to achieve high accuracy in the UWB indoor positioning system, it is imperative to mitigate the impact of NLOS propagation [7,10]. There are many methods to mitigate the impact of NLOS propagation on UWB positioning. Common approaches include directly optimizing the filtering framework (e.g., Bayesian, Kalman filtering, etc.) [1,11], and preprocessing ranging data through NLOS detection to remove or attenuate NLOS components before feeding these data into the filter [12]. The latter has become the most widely used method in engineering due to its high compatibility with classical filtering algorithms. Accurate identification of NLOS propagation is a key prerequisite for overcoming the non-Gaussian nature of ranging errors and thereby improving UWB positioning accuracy in complex environments. Therefore, NLOS identification is crucial for UWB positioning systems [13].

In early studies, NLOS identification was performed based on manually extracted statistical features from signals rather than using Machine Learning (ML) algorithms [14,15]. However, this type of NLOS identification algorithm is highly dependent on manual feature design capabilities. If the features are unreasonably designed, it will lead to low recognition accuracy. Additionally, the core parameters of the threshold method and feature fusion decision (such as thresholds and feature weights) need to be manually optimized based on experimental data from specific scenarios, resulting in poor environmental adaptability. The NLOS identification method based on ML utilizes the automatic feature learning ability of the model to directly capture the essential differences between LOS and NLOS from the original signal or low dimensional features, providing a new path for accurate recognition in complex scenes. At present, common NLOS recognition methods are mainly based on ML techniques, which can be classified into two categories: one is based on collected raw physical data (e.g., raw Channel Impulse Response (CIR)), and the other is the feature-based method [7]. The core principle of ML-based NLOS identification using raw CIR is to leverage ML models to automatically extract the differential features between Line-of-Sight (LOS) and NLOS propagation from raw CIR signals, thereby realizing the classification of these two propagation scenarios. Jiang et al. [16] developed a UWB NLOS/LOS signal classification approach based on Long Short-Term Memory (LSTM) and Convolutional Neural Network (CNN). Authors adopted CNN to automatically extract features, and then fed the CNN output into the LSTM to perform identification. Liu et al. [17] proposed an NLOS/LOS classification strategy employing CNN and Gated Recurrent Unit (GRU). Authors utilized the CNN and GRU to capture spatial features and temporal features, respectively. With squeeze-and-excitation blocks embedded in these architectures, researchers are able to weight channel-wise features adaptively. The core principle of feature-based ML for NLOS identification is to first manually design or computationally extract key features that can distinguish between LOS and NLOS from signals. Then, ML models are employed to capture the relationship between these features and propagation scenarios, and, finally, the classification is realized. Si et al. [13] developed a NLOS identification strategy that leverages CNN-derived features from raw CIR and handcrafted features to distinguish NLOS from LOS conditions. In the developed method, the Multilayer Perceptron (MLP) was employed to fuse the CNN-extracted features with six manual features. The results illustrate that the presented approach achieves superior performance over the traditional image-based CNN method, with a performance improvement of 44.16%. Yang et al. [18] put forward a two-step identification approach for classifying UWB channels, which relies on the fuzzy credibility-based Support Vector Machine (SVM) and dynamic threshold comparison. Although these existing NLOS identification methods can achieve a certain level of identification performance in their verified scenarios, these approaches exhibit strong scene dependency. When the test environment changes (e.g., switching from an indoor office scenario to a complex outdoor environment, or from a single-obstacle scenario to a multi-obstacle scenario), their recognition accuracy will decrease significantly due to differences in the distribution of environmental features.

To address this critical issue, in recent years, some scholars have proposed integrating transfer learning (TL) algorithms into the NLOS identification framework. By exploring the conventional features of NLOS signals across various scenarios and mitigating the impact of distribution shifts caused by scenario variations, this approach offers a novel solution to the issue of unstable recognition accuracy in cross-scenario NLOS applications. Sun et al. [19] observed that integrating TL into the Stockwell transform and convolutional neural network (ST-CNN)-based NLOS identification approach can effectively cut down both training time and data requirements compared to the standalone ST-CNN method, while still preserving high performance levels. Fontaine et al. [7] proposed a TL strategy for UWB NLOS identification employing feature-based and CIR-based NNs. For the feature-based method, the Deep Neural Network (DNN) was used to identify NLOS. For the raw CIR-based method, the CNN was used to identify NLOS. In addition, the authors put forward automatic optimization strategies for TL-based DNNs, enabling these models to adapt to new environments and different UWB configurations. Nkrow et al. [20] provided a robust TL-based UWB NLOS identification method, leveraging CIR data collected from two separate environments. Park et al. [21] introduced a TL framework for UWB NLOS/LOS recognition, employing MLP and CNN as classifiers in an unmeasured scenario. The developed approach delivers about 10% improvement in accuracy and accelerates the training process by nearly five times. Subsequently, scholars such as Wang [22] found through their research that compared with transfer learning technology, the introduction of Domain-Adversarial Neural Network (DANN) can achieve higher NLOS identification accuracy. Wang et al. [22] developed a novel enhanced DANN for recognizing UWB signal occlusion in dynamic environments. The developed NLOS recognition strategy utilizes CIR and manual CIR features. The experimental results show that in binary/multi-class classification and environmental transfer scenarios, the DANN-based NLOS identification accuracy is significantly superior to that of traditional Machine Learning (i.e., Binary Hypothesis Testing, SVM), deep learning (i.e., CNN), and basic transfer learning methods. Results demonstrate that the developed strategy attains over 97.36% accuracy in traditional LOS/NLOS binary classification across different scenarios, with multi-class classification accuracy reaching 97.07%. However, in [22] the handcrafted features do not require additional processing by deep learning algorithms (such as convolutional layers or fully connected layers); instead, they are fused with CIR features solely through concatenation. In addition, the feature extractor employs four convolutional layers and four pooling layers in [22]. While this method effectively extracts deep features of CIR signals, its ability to capture temporal information of signals in dynamic environments is limited.

To address this shortcoming, we develop a novel NLOS identification strategy based on a Domain-Adversarial Neural Network for UWB positioning systems. In the proposed NLOS identification strategy, the DANN framework is used to significantly boost its cross-domain adaptation capability, with the CNN-DAE-MLP-Attention (CDM) method utilized for feature extraction. Specifically, we use a 1D CNN to capture local and temporal features from the raw CIR. The Denoising Autoencoder (DAE) is adopted to denoise and reconstruct the original handcrafted features. The handcrafted features processed by DAE are fed into the MLP for further feature extraction. Then, the attention mechanism is used to fuse the features extracted by CNN and MLP. Finally, the fused features are fed to the label predictor and domain classifier, respectively; the former is for NLOS identification with supervised training, while the latter is employed for adversarial training to learn domain-invariant features by distinguishing the source and target domains. Numerous parameters need to be tuned in the NLOS identification process. However, manual parameter tuning is excessively time-consuming, and the trial-and-error process is prone to limitations imposed by experience, making it difficult for parameter combinations to cover the optimal solution. This compromises the performance stability and generalization capability of NLOS recognition tasks. Therefore, we introduce an automated hyperparameter tuning strategy for the proposed method using Bayesian optimization to resolve this series of problems. We also compare the proposed method with other methods in diverse environments. The key contributions of this paper are summarized as follows.

1. We develop a novel strategy combining CDM with DANN (CDMD) for UWB LOS/NLOS signal identification. The proposed CDMD method not only has cross-scenario adaptability, but is also robust in identifying UWB NLOS signals.
2. We propose a novel feature extraction approach: on the one hand, it automatically extracts CIR features using the CNN network; on the other hand, it performs deep processing on manually designed features through DAE and MLP. The proposed approach leverages the denoising and reconstruction mechanism of DAE to effectively filter out noise and interference from the manually extracted features by learning the mapping relationship that recovers authentic features from noisy ones, thereby enhancing the quality and robustness of the features.

The remainder of this article is organized as follows. In Section 2, we describe the background theoretical knowledge regarding the devised approach. Then, we present the developed CDMD approach for NLOS identification in Section 3. In Section 4, we present a detailed discussion on the experimental setup and the analysis of the experimental results. Finally, the conclusions are presented in Section 5.

## 2. Theoretical Framework

### 2.1. Signal Model

In the UWB localization system, signal propagation between two devices may be affected by the transmission environment. When the signal penetrates obstacles or undergoes reflection, it can cause transmission distortion. The CIR can effectively capture and reflect these issues. In NLOS identification, the CIR is an extremely important and fundamental feature. The transmitted signal s(t) of each anchor point is modeled as [16]

(1) $$s\left(t\right)=\sum_{i=-\infty }^{\infty }\sum_{j=0}^{N_{c}-1}\alpha_{j}q\left(t-iT_{s}-jT_{c}\right)$$

where q(t) denotes the single Gaussian pulse with repetition time $T_{c}$, a symbol duration $T_{s}$ consists of $N_{c}$ pulses, and $\alpha_{j}\in \left\{-1,+1\right\}$ denotes the polarization sequence for the spectrum shaping. In this work, we consider a linear time-invariant (LTI) UWB channel, where the CIR can be modeled as [16,23]

(2) $$h\left(t\right)=\sum_{l=1}^{L}\eta_{l}\delta \left(t-\tau_{l}\right),$$

where $\eta_{l}$ denotes the fading coefficients of the *l*-th path, δ(·) denotes the Dirac pulse function, *L* denotes the number of paths, and $\tau_{l}$ denotes the time delay of the *l*-th path.

In this work, we consider a LTI UWB multipath channel. Under this assumption, the received signal r(t) is a superposition of delayed and attenuated copies of the transmitted signal s(t) from different paths, plus additive noise, as given by [16,23]

(3) $$r\left(t\right)=\sum_{l=1}^{L}\eta_{l}s\left(t-\tau_{l}\right)+w\left(t\right),$$

where w(t) denotes the additive white Gaussian noise and s(t) is the transmitted UWB signal.

### 2.2. Feature Calculation

In this work, we aim to identify NLOS and LOS conditions based on the measured raw CIR and manually extracted channel features, specifically, total energy (TE), strongest power energy (SPE), and Root Mean Square Delay Spread (RMS-DS), mean excess delay (MED), skewness, kurtosis, K-factor, Signal-to-Noise Ratio (SNR), rise time (RT), the ratio of the first component power to the total energy, and the ratio of the second amplitude sample of the first-path detector to the total energy.

#### 2.2.1. Channel Impulse Response

The CIR is the essential representation of the channel, encapsulating comprehensive information about the communication link condition. It is commonly adopted as the direct output in channel measurement systems or channel estimation modules, without the necessity of additional feature extraction [9]. Equation (2) presents the CIR model in the continuous delay domain.

#### 2.2.2. Total Energy

TE can be calculated by [24]

(4) $$E=\sum_{l=1}^{L}{\left|\eta_{l}\right|}^{2},$$

where $\eta_{l}$ denotes the fading coefficients of the *l*-th path, *L* denotes the number of paths, and |·| represents the magnitude operator.

#### 2.2.3. Strongest Power Energy

The strongest power energy can be calculated by [25]

(5) $$\varepsilon_{SPE}=\max_{1\leq l\leq L}{\left|\eta_{l}\right|}^{2}.$$

#### 2.2.4. Root Mean Square Delay Spread

The RMS-DS describes the temporal dispersion of wireless channels. It can be calculated by

(6) $$\tau_{rms}=\sqrt{\frac{\sum_{l=1}^{L}{\left(\tau_{l}-\tau_{med}\right)}^{2}\cdot {\left|\eta_{l}\right|}^{2}}{\sum_{l=1}^{L}{\left|\eta_{l}\right|}^{2}}}$$

where *l* is the index of the path, *L* denotes the number of paths, $\tau_{l}$ denotes the delay of the *l*-th path, and $\eta_{l}$ denotes the fading coefficients of the *l*-th path. $\tau_{med}$ represents the mean excess delay, which is written as

(7) $$\tau_{med}=\frac{\sum_{l=1}^{L}\tau_{l}{\left|\eta_{l}\right|}^{2}}{\sum_{l=1}^{L}{\left|\eta_{l}\right|}^{2}}$$

#### 2.2.5. Skewness

In the channel, skewness measures the asymmetry of the CIR Power Delay Profile (PDP) or received signal distribution. In LOS channels, the direct path is usually the strongest and arrives first. Most of the received power is concentrated at the beginning of the delay profile, while later multipath components (reflections, scattering) are weaker. In NLOS channels, there is no direct dominant path. Received energy is spread across multiple delayed components, sometimes with significant late arrivals. Skewness values in LOS are often smaller in magnitude compared to NLOS. The calculation of skewness is expressed as

(8) $$S=\frac{1}{L}\sum_{l=1}^{L}{\left(\frac{\left|\eta_{l}\right|-\mu }{\sigma }\right)}^{3},$$

where μ denotes the mean and $\mu =\frac{1}{L}\sum_{l=1}^{L}\left|\eta_{l}\right|$. σ demotes the standard deviation, $\sigma =\sqrt{\frac{1}{L}\sum_{l=1}^{L}{\left(\left|\eta_{l}\right|-\mu \right)}^{2}}$.

#### 2.2.6. Kurtosis

Under LOS propagation conditions, the multipath energy distribution is more concentrated, with the main energy focused on the dominant path, resulting in a larger kurtosis of the CIR PDP. In contrast, under NLOS conditions, the multipath energy distribution is more dispersed, leading to a relatively smaller kurtosis of the CIR power delay profile. Kurtosis can be written as [22,24]

(9) $$K_{ur}=\frac{1}{L}\sum_{l=1}^{L}{\left(\frac{\left|\eta_{l}\right|-\mu }{\sigma }\right)}^{4}.$$

#### 2.2.7. K-Factor

The Ricean K-factor is commonly employed to characterize the severity of small-scale fading by describing the power ratio between the dominant LOS component and the scattered multipath components. It is written as

(10) $$K=\frac{P_{LOS}}{P_{sca}},$$

where $P_{LOS}$ represents the power of the dominant LOS component. In our work, $P_{LOS}$ is defined as the power of the strongest path in the CIR, which is given by

(11) $$P_{\mathrm{LOS}}=\max_{1\leq l\leq L}{\left|\eta_{l}\right|}^{2}.$$

$P_{sca}$ denotes the power of the scattered components, which is calculated as

(12) $$P_{sca}=\sum_{l=1}^{L}{\left|\eta_{l}\right|}^{2}-P_{\mathrm{LOS}}.$$

#### 2.2.8. Signal-to-Noise Ratio

SNR reflects the ratio between signal strength and noise intensity. In LOS scenarios, the received signal power is relatively high, path loss is minimal, and thus the SNR is relatively large. In NLOS scenarios, the signal undergoes obstruction, reflection, and diffraction, increasing multipath loss and reducing received power. In NLOS conditions, there is more noise, thereby causing a reduction in SNR. SNR can be written as

(13) $$SNR=\frac{P_{s}}{P_{n}},$$

where $P_{n}$ denotes the noise power, $P_{n}=E\left[{\left|w\left(t\right)\right|}^{2}\right]$, E[·] denotes the expectation operator, and w(t) denotes the additive white Gaussian noise. $P_{s}$ denotes the average power of the received signal, which is given by

(14) $$P_{s}=P_{tx}\cdot \sum_{l=1}^{L}{\left|\eta_{l}\right|}^{2},$$

where $P_{tx}$ is the average power of the transmitted signal s(t) and $P_{tx}=E\left[{\left|s\left(t\right)\right|}^{2}\right]$. The SNR can be expressed in decibels (dB), $SNR_{dB}=10log_{10}\frac{P_{s}}{P_{n}}$.

#### 2.2.9. Rise Time

The rise time is defined as the time interval between the instant when the cumulative CIR energy reaches 10% of the total energy and the instant when it reaches 90% [24]:

(15) $$RT=\tau_{stop}-\tau_{start},$$

where $\tau_{stop}$ denotes the time instant at 90% of the total cumulative energy and $\tau_{start}$ denotes the time instant at 10% of the total cumulative energy.

#### 2.2.10. The Ratio of the First Component Power to the Total Energy

The ratio of the first component power to the total energy is defined as

(16) $$R=\frac{P_{f}}{E},$$

where $P_{f}$ denotes the power of the first component extracted from the CIR signal, *E* denotes the sum of the powers of all multipath components in the CIR, i.e., the total energy of the CIR, calculated as $E=\sum_{l=1}^{L}{\left|\eta_{l}\right|}^{2}$. In addition, the ratio of the second amplitude sample of the first-path detector to the total energy is adopted in this paper, where the second amplitude sample of the first-path detector can be directly obtained from UWB devices.

## 3. Proposed NLOS/LOS Identification Method

**Figure 1:**
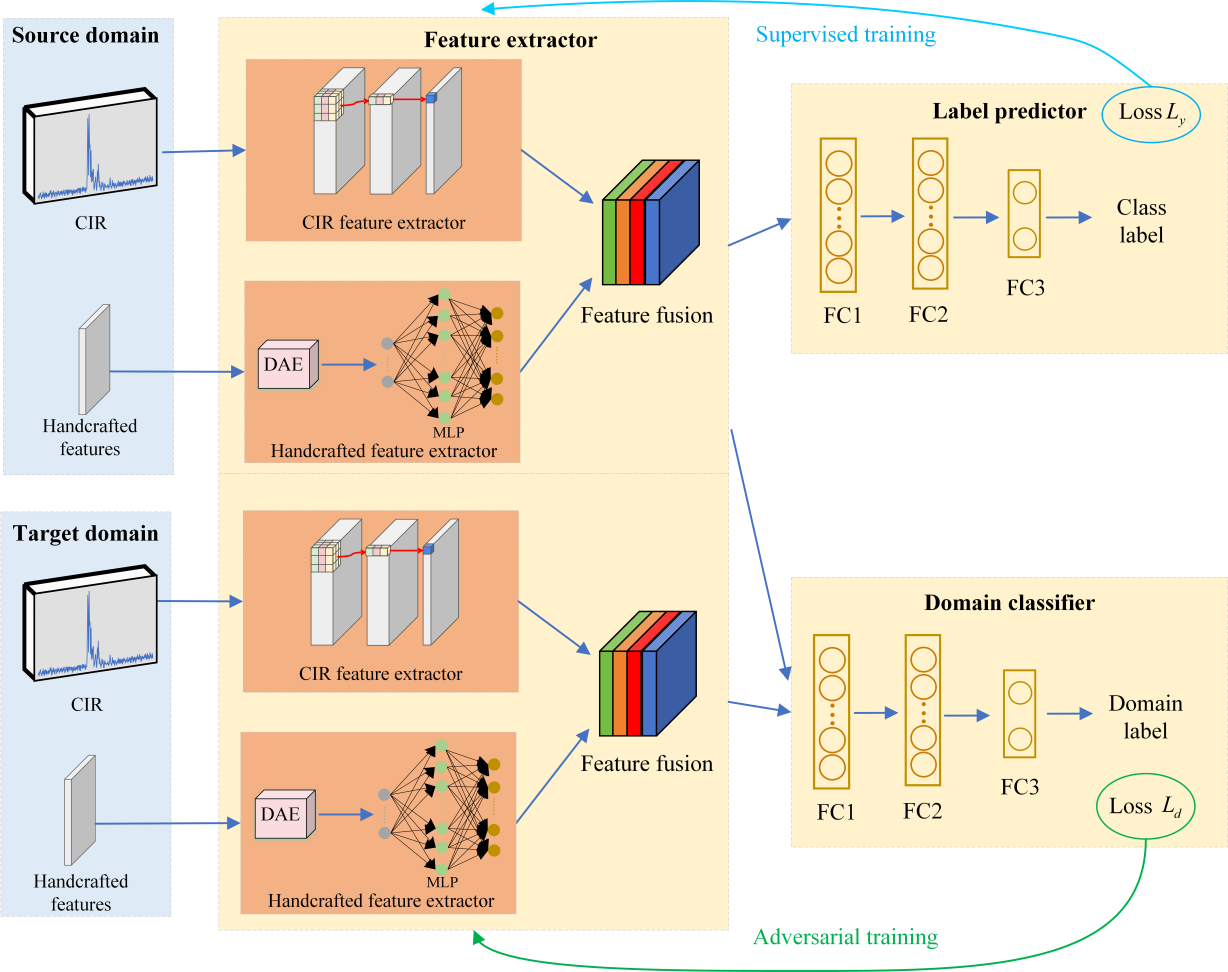
The framework of the CDMD method.

DANN introduces the concept of domain-adversarial learning. The DANN employs adversarial training to explicitly minimize the distribution discrepancy between the source and target domains, thereby improving the generalization ability of the model to unseen scenarios [22]. Based on the DANN framework [26], we develop a novel CDMD approach to classify LOS and NLOS conditions. Figure 1 presents the overall framework of the developed NLOS recognition method in this paper. The basic structure of the proposed method comprises three modules, namely, the label predictor, domain classifier, and feature extractor. In the developed identification strategy, the feature extractor module mainly captures high-level features from the input CIR data and handcrafted features. The label predictor employs the derived features for LOS/NLOS discrimination. The extracted features are first fed into the Gradient Reversal Layer (GRL), which acts as an identity transformation during forward propagation to preserve feature representations for the subsequent domain classifier. During backpropagation, the gradient of the domain classification loss is multiplied by a negative coefficient via the GRL, thereby inverting the gradient signal before it propagates back to the shared feature extractor. The GRL-enabled adversarial training forces the feature extractor to learn domain-invariant features, while the label predictor (trained only on source-domain samples) learns task-relevant features for NLOS/LOS classification. The role of the domain classifier is to distinguish whether the features are derived from the target domain or the source domain.

### 3.1. Feature Extractor

Using a single type of feature for NLOS identification makes it difficult to achieve high accuracy. To enhance LOS/NLOS identification performance, we employ both the measured CIR features and manually extracted features for NLOS identification. In this paper, we present a novel feature extraction framework leveraging CNN and DAE-MLP. The feature extraction architecture primarily consists of the CIR feature extractor, the handcrafted feature extractor and feature fusion module. The CIR feature extractor is designed to learn discriminative patterns from the CIR. It employs a 1D CNN for temporal feature extraction. The handcrafted feature extractor consists of DAE and MLP. Most existing feature extraction methods directly employ deep learning modules to process handcrafted features, or fuse handcrafted features with other types of features for NLOS identification. However, handcrafted features typically contain environmental noise and measurement errors, especially under NLOS conditions, which significantly degrade feature quality and recognition reliability. To effectively suppress noise interference in handcrafted features and learn more robust representations, the DAE is adopted in this paper to denoise and reconstruct the original handcrafted features. The handcrafted features processed by DAE are fed into the deep learning module for further feature extraction, thereby improving the accuracy and stability of NLOS identification. Specifically, the manually extracted features are used as the input of the DAE. The high-order nonlinear features generated by the DAE are fed into the MLP, which maps these features into an optimized feature space via multiple nonlinear activation functions (e.g., ReLU). Finally, we leverage an attention mechanism to perform weighted fusion of the features extracted by the CNN and DAE-MLP modules, generating a robust shared feature representation. Subsequently, this shared representation is fed into both a label predictor for NLOS identification and a domain classifier for domain classification.

#### 3.1.1. CIR Feature Extractor

Since CNNs are effective at capturing local correlations and spatial patterns, they can extract local features of the CIR in the delay domain and are therefore widely used for NLOS classification tasks. 1D CNN are frequently employed for time series data, as they can perform convolutions directly along the temporal dimension to capture local temporal patterns. Therefore, this paper utilizes a 1D CNN to extract temporal features from CIR samples.

A typical CNN consists of three main layers, namely, convolutional layers, pooling layers, and fully connected (FC) layers [13,27,28]. In this paper, the input data of the CNN is CIR data. The convolutional layer employs convolutional kernels (filters) to extract local features and capture local correlations within the data. Pooling layers are usually added after convolutional layers to reduce computational load while preserving key features. The FC layer integrates the features extracted by the convolutional and pooling layers, mapping the convolutional features to a low-dimensional vector whose length equals the number of categories. The convolutional layers and FC layers (except the final layer) are sometimes followed by a ReLU activation function. By applying the ReLU activation after the convolutional layers, nonlinearity is introduced, which helps mitigate the vanishing gradient problem and accelerates convergence. By employing ReLU after the intermediate fully connected layers, the nonlinear expressive capability of features prior to classification is enhanced.

#### 3.1.2. Handcrafted Feature Extractor

We extract a series of handcrafted features from the CIR, however, measurement noise exists in the manual features. DAE can eliminate noise and redundant information from high-dimensional spaces [29]. We employ DAE to filter noise from manual features through encoding–decoding reconstruction constraints. In addition, employing DAE enables the capture of nonlinear characteristics within manually extracted features, and their transformation into higher-order features with stronger discriminability. The DAE is trained by minimizing the reconstruction loss, which ensures the encoded features retain the critical information of the original static features. After DAE processing, the feature boundaries of NLOS/LOS become clearer when input into the MLP, thereby enhancing NLOS recognition accuracy.

The MLP learns the importance of different features, automatically assigning higher weights to more discriminative ones, while also capturing nonlinear interactions among features. An MLP comprises an input layer, hidden layers, and a final output layer [21]. The input layer is designed to have a neuron count equal to the feature dimension. Typically, one to three hidden layers are used, as too many layers may lead to overfitting. The output layer dimension is consistent with the dimension of the high-dimensional feature vector. In our work, manually extracted features are first fed into a DAE to eliminate noise and redundant information, and the resulting low-dimensional representations are then input to the MLP, which outputs the final feature vectors for these preprocessed manual features.

#### 3.1.3. Feature Fusion

In this paper, we consider two separate feature streams. One feature stream consists of the temporal dynamic features extracted by the CNN, with raw CIR data serving as the input to the CNN. These features characterize the intrinsic time-domain properties of the wireless channel. The other feature stream comprises the denoised handcrafted features optimized by the MLP. These two feature types possess distinct characteristics and information content. If we simply concatenate the features, we may fail to fully leverage their respective importance. To enhance the accuracy of NLOS identification, the attention fusion mechanism is designed to adaptively integrate two separate feature streams for NLOS/LOS classification. The attention fusion mechanism adaptively learns task-specific attention weights for each feature stream within two heterogeneous feature streams, rather than manually assigning fixed weights. First, it assigns a dedicated adaptive weight to the CNN-derived temporal dynamic feature stream and the MLP-optimized denoised handcrafted feature stream, respectively. These weights are learned to quantify the discriminative contribution of each feature stream to the NLOS/LOS classification task. Then, the two feature streams are concatenated in a weighted summation manner based on the learned adaptive weights. This adaptive weighting strategy highlights the more informative feature stream for NLOS/LOS discrimination while suppressing the irrelevant one, thereby enhancing the discriminability of the fused features. This approach fully leverages the statistical information from manual features and the dynamic information from temporal features.

### 3.2. Domain Classifier

The domain classifier mainly serves to discriminate whether features derived from the feature extractor belong to the target domain or the source domain. During training, the domain classifier updates its parameters by minimizing the domain classification loss (e.g., cross-entropy loss) for source- and target-domain discrimination. Meanwhile, a gradient reversal layer is inserted between the shared feature extractor and the domain classifier. This layer inverts the domain classification loss gradient by multiplying it with a negative coefficient during backpropagation before the gradient is propagated back to the feature extractor. This adversarial mechanism forms a minimax game between the feature extractor and the domain classifier, driving the feature extractor to capture domain-invariant and task-relevant features that the domain classifier cannot identify. This effectively aligns the feature distributions of the source and target domains, and improves the generalization ability of the model on the unlabeled target domain for NLOS recognition.

The target-domain samples have no LOS/NLOS labels and only carry domain labels, therefore only the domain classification loss is calculated for them in the training process. The loss function of the domain classifier is written as [22]

(17) $$L_{d}=-\frac{1}{M_{s}}\sum_{i=1}^{M_{s}}\log\left(1-G_{d}\left(f_{s}^{\left(i\right)}\right)\right)-\frac{1}{M_{t}}\sum_{j=1}^{M_{t}}\log\left(G_{d}\left(f_{t}^{\left(j\right)}\right)\right)$$

where $G_{d}\left(\cdot \right)$ represents the predicted probability of belonging to the 1 class for the *i*-th sample, which is obtained from the second dimension of the softmax-normalized 2-dimensional network output. In domain classifier, 1 denotes the target domain and 0 denotes the source domain. In Formula (17), $G_{d}\left(f_{s}^{\left(i\right)}\right)$ denotes the probability that the domain predictor predicts the source-domain feature $f_{s}^{\left(i\right)}$ as the target domain. $G_{d}\left(f_{t}^{\left(j\right)}\right)$ denotes the probability that the domain predictor predicts the target-domain feature $f_{t}^{\left(j\right)}$ as the target domain. $M_{t}$ and $M_{s}$ represent the numbers of training samples in the target domain and source domain, respectively.

### 3.3. Label Predictor

Based on the features generated by the feature extractor, the label predictor is designed to distinguish between LOS and NLOS UWB propagation states. The label predictor learns classification through features solely from the source domain and is updated by minimizing the label prediction loss (e.g., cross-entropy loss). In this work, the loss function utilizes cross-entropy between actual and predicted labels. During the training process, the parameters of the label predictor (such as the weights and biases parameters of the fully connected layer) and feature extractor are continuously updated to better distinguish between NLOS and LOS, relying on the features output by the feature extractor.

The training data $\chi ={\left\{x_{i},y_{i}\right\}}_{i=1}^{M_{s}}$ is given, where $M_{s}$ denotes the number of source-domain training samples and $y_{i}\in \left\{0,1\right\}$. In label predictor, the true label $y_{i}\in \left\{0,1\right\}$ is defined such that $y_{i}=0$ indicates the LOS scenario and $y_{i}=1$ denotes the NLOS scenario. In the case of two-class classification (LOS/NLOS), the loss function is written as [22,30]

(18) $$L_{y}=-\frac{1}{M_{s}}\sum_{i=1}^{M_{s}}\left[\left(1-y_{i}\right)\log\left(1-G_{y}\left(f_{s}^{\left(i\right)}\right)\right)+y_{i}\log\left(G_{y}\left(f_{s}^{\left(i\right)}\right)\right)\right]$$

where $M_{s}$ denotes the number of training samples in the source domain, $y_{i}$ denotes the true label of the *i*-th sample, $G_{y}\left(f_{s}^{\left(i\right)}\right)$ denotes the predicted probability of belonging to the 1 class for the *i*-th sample. In Formula (18), $G_{y}\left(f_{s}^{\left(i\right)}\right)$ specifically represents the label predictor’s predicted probability of the *i*-th sample belonging to the NLOS class, which is obtained from the second dimensional of the softmax-normalized 2-dimensional network output. During the prediction, for a given test sample *x*, the output of the identification is performed using $G_{y}\left(f_{s}^{\left(i\right)}\right)$. If $G_{y}\left(f_{s}^{\left(i\right)}\right)<0.5$, it is categorized as LOS; otherwise, it is classified as NLOS. Note that the loss for the label predictor is calculated only on the source domain, as only the source domain possesses true label values for LOS/NLOS. Target-domain samples lack labels and do not contribute to the calculation of the label predictor loss.

To realize the domain-adversarial training and balance the task performance (NLOS classification) and domain generalization ability, the total loss function of the CDMD model is constructed by combining the classification loss of the label predictor, the domain classification loss of the domain classifier, and the reconstruction loss of DAE. The parameters λ and β are used to adjust the weights for the domain-adversarial loss and the DAE reconstruction loss, respectively. The overall loss function is calculated by

(19) $$L=L_{y}-\lambda L_{d}+\beta L_{dae}$$

where $L_{y}$ denotes the label classification loss, $L_{d}$ denotes the domain classification loss, and λ represents a regularization parameter that prevents the trained network from overfitting. β denotes the weight of the DAE reconstruction loss and $L_{dae}$ denotes the DAE reconstruction loss and is calculated by

(20) $$L_{dae}=\frac{1}{M_{s}}\sum_{i=1}^{M_{s}}{∥x_{\mathrm{static},s}^{\left(i\right)}-\mathrm{DAE}\left(x_{\mathrm{static},s}^{\left(i\right)}\right)∥}_{2}^{2}+\frac{1}{M_{t}}\sum_{j=1}^{M_{t}}{∥x_{\mathrm{static},t}^{\left(j\right)}-\mathrm{DAE}\left(x_{\mathrm{static},t}^{\left(j\right)}\right)∥}_{2}^{2}$$

where $M_{s}$ denotes the number of training samples in the source domain and $M_{t}$ represents the numbers of training samples in the target domain. $x_{\mathrm{static},s}^{\left(i\right)}$ represents the *i*-th static feature vector from the source domain, $x_{\mathrm{static},t}^{\left(j\right)}$ denotes the *j*-th static feature vector from the target domain, and DAE(·) denotes the DAE function that maps input features to reconstructed outputs. ${∥\cdot ∥}_{2}^{2}$ represents the squared L2 norm.

During model training, the domain classifier is independently optimized by minimizing $L_{d}$ (with feature extractor and label predictor parameters fixed), enabling it to accurately discriminate domain origins. The label predictor and shared feature extractor are jointly optimized by minimizing the total loss L (with domain classifier parameters fixed). This forces the feature extractor to simultaneously satisfy two conflicting objectives. These objectives are minimizing $L_{y}$ to preserve classification discriminability and maximizing $L_{d}$ to confuse the domain classifier, while the DAE module enhances static feature denoising via the reconstruction loss $L_{\mathrm{dae}}$.

### 3.4. DANN Optimization Strategy

Manual hyperparameter tuning is not only time-consuming and labor-intensive but also prone to limitations imposed by human experience, making it difficult for parameter combinations to cover the global optimal solution. In addition, repeated testing of different hyperparameter values consumes substantial computational resources, reduces model iteration efficiency, and may even lead to unreasonable parameter configurations due to subjective judgment biases, thereby affecting the performance stability and generalization ability of the NLOS recognition. Therefore, we employed Bayesian optimization to determine the optimal combination of hyperparameters, comprising dropout rate, learning rate, the number of filters per layer in CNN, the number of convolution layers in CNN, the number of neurons in each layer of the MLP, and the number of hidden layers in MLP, etc. In this study, we adopted the prediction accuracy on the validation set as the objective function for the Bayesian optimization process.

First, we defined the search space of the hyperparameters to be optimized. The search space is configured as follows: the learning rates of the domain predictor, label predictor, and feature extractor are set to the range of [0.0001, 0.01]; the dropout rates are set to the range of [0.3, 0.5]. The Bayesian optimization process is conducted with the following settings: we set a batch size of 32, adopt Adam as the optimizer for all models, and fix the initial training epochs to 250. Based on the above fixed experimental settings, we specifically configured the Bayesian optimizer to perform a hyperparameter search by maximizing the objective function. We first performed a random search with 10 initial points to explore the hyperparameter space broadly, followed by 90 Bayesian optimization iterations for targeted search. In total, 100 hyperparameter combinations were sampled and evaluated. Each hyperparameter combination sampled by Bayesian optimization was trained independently with a fixed seed (42), and the validation accuracy was taken as the objective function value for that combination. The Bayesian optimization process terminated when the preset 100 Bayesian optimization iterations were completed.

After obtaining the optimal learning rates and dropout rates from the Bayesian optimization, we independently configured the network structure of the model. Specifically, we selected the optimal network structure by evaluating the model performance under different structure configurations with the optimized hyperparameters. The search space for the network structure was defined as discrete values: the CNN component of the feature extractor is set to 2 or 3 convolutional layers, with the number of filters in each convolutional layer selected from [32, 64, 128]; the MLP component of feature extractor is configured with 1 to 3 hidden layers, and the hidden layer dimensions are also selected from [8, 16, 32]. The final network structure was determined based on the highest validation accuracy achieved during the evaluation of these configurations.

Finally, we fine-tuned the optimized hyperparameters. We adopted a fixed-step learning rate decay strategy during training, where the learning rate was reduced by 30% every 120 epochs (starting from the optimal initial learning rate obtained from the first Bayesian optimization stage). The final optimal hyperparameter configuration, including the initial learning rates, dropout rates, network structure, and learning rate decay details, is presented in Table 1 and Table 2.

## 4. Results and Discussion

### 4.1. Experimental Setup and Data Collection

The experimental UWB platform was developed based on the Decawave DW1000 chip (now Qorvo) [31]. Using Decawave DW1000-based UWB platform, we collected CIR data to enable high-precision ranging measurements. The employed UWB system is capable of the real-time transmission of parameter data to the computer acquisition software. In the measurement campaign, the anchor node and the tag were mounted on tripods, with the tag responsible for transmitting signals and the anchor node for receiving signals. The anchor node was linked to a laptop through a data cable to record the collected data in real time. The tag transmitted signal at the center carrier frequency of 3.5 GHz with a measurement bandwidth 500 MHz. The employed UWB system was configured with a transmitted signal power spectral density of –18 dBm/MHz. Its effective communication distance is 600 m in the open LOS environment. The transmit and receive antennas were both omnidirectional. Across all scenarios, the experimental setup remained consistent to ensure the reproducibility and comparability of the results.

**Figure 2:**
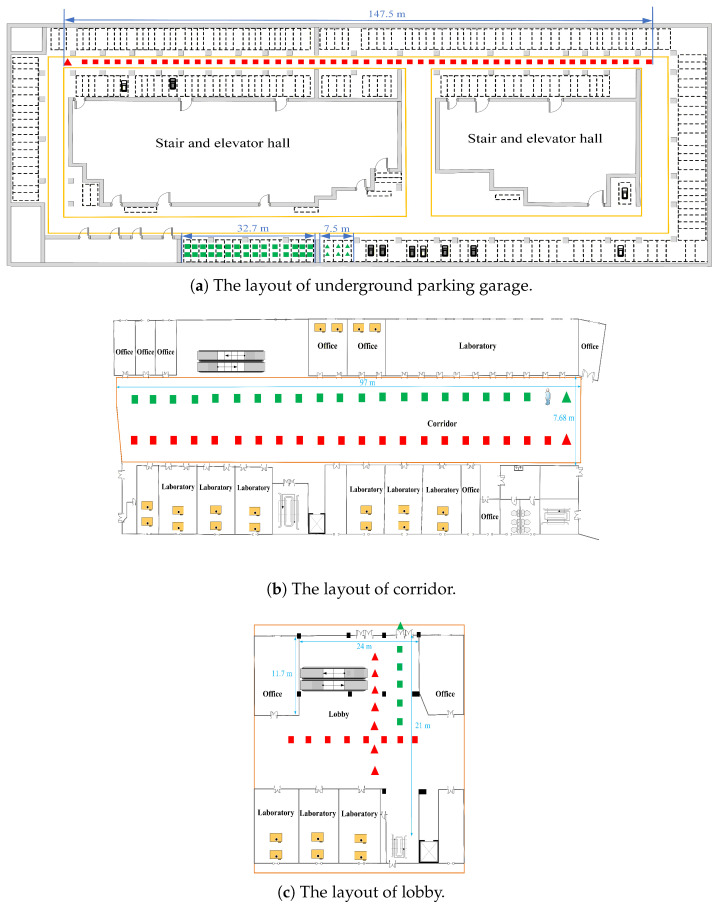
The layout of experimental environments.

**Figure 3:**
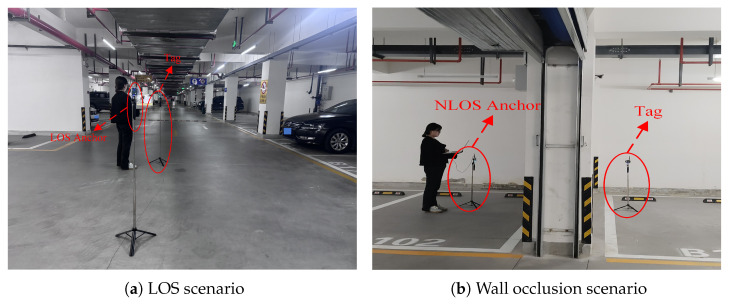
The underground parking garage environment.

The measurement campaigns were performed in three scenarios (i.e., underground parking garage, corridor, and lobby). The floor plan of experimental environments is shown in Figure 2. The deployment positions of anchors (marked with triangles) and tags (marked with squares) are indicated in Figure 2, with the measurement range under LOS conditions shown in red and that under NLOS conditions shown in green, respectively. First, we conducted measurement activities in a real underground parking garage. We denote the underground parking garage scenario as Scenario A. Measurements were conducted in typical scenarios including unobstructed LOS and wall obstruction. In LOS scenarios, the tag and anchor were deployed in an open area of the underground parking garage, as shown in Figure 3a. In NLOS scenarios, the tag and anchor were obstructed by a concrete wall, as shown in Figure 3b. Figure 3b demonstrates the occlusion of the NLOS anchor and tag caused by a concrete wall. During the measurement process, we placed the anchor node at a random fixed position and moved the tag throughout the environment. We collected measurements for LOS and wall occlusion scenarios.

**Figure 4:**
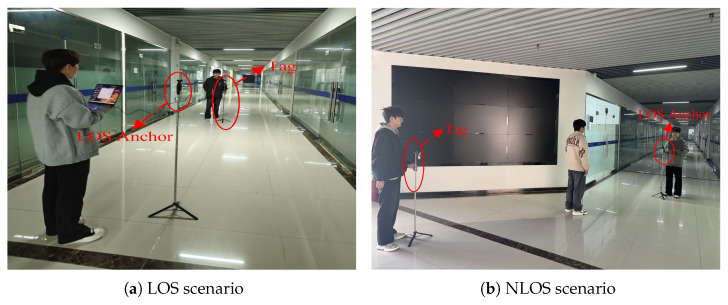
The corridor environment.

Subsequently, we conducted similar measurement activities in the corridor. The corridor is denoted as Scenario B. The experimental setup for the LOS scenario is relatively simple, with tag and LOS anchor placed in open regions of the corridor. For the NLOS scenario in the corridor, a human was employed as the obstacle. Figure 4a,b demonstrate the LOS scenario and the human occlusion scenario in the corridor.

**Figure 5:**
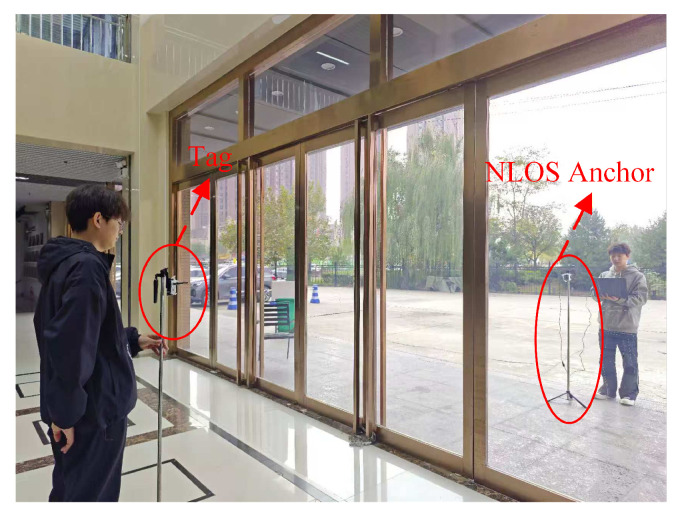
The lobby environment.

Moreover, we conducted measurement activities in the lobby. The lobby is denoted as Scenario C. For LOS environments, the anchor and the tag were placed in the lobby, with a distance of over 1 m between them. For the NLOS scenario in the lobby, the glass door was employed as the obstacle. In the NLOS scenario, the signal propagates through a glass door, where the anchor and tag were alternatively placed inside and outside the door for different experimental groups. Figure 5 demonstrates the glass door occlusion scenario in the lobby. For each measurement, the dataset contains the estimated range, Received Signal Strength (RSS), noise standard deviation, maximum noise level and CIR, etc. In each scenario, we collected 7200 samples, comprising 3500 samples for LOS and 3700 samples for NLOS.

### 4.2. NLOS Identification Performance

In this paper, we employ metrics such as accuracy, precision, recall and F1-score to assess the performance of NLOS identification [10,32].

#### 4.2.1. NLOS Identification Performance of Different Algorithms

We carried out comparative experiments on NLOS recognition based on three distinct scenarios: underground parking garage, corridor and lobby. We compared the proposed CNN-DAE-MLP-Attention (CDM) method with CNN-Attention, MLP-Attention, and CNN-MLP-Attention algorithms. The CDM method (CDMD without DANN) is the proposed method, excluding the DANN. For each environment, we adopt 70% of the samples as the training set, 10% of the samples as the validation set, and 20% of the samples as the test set. Specifically, 5040 samples are used as the training set, including 2450 LOS samples and 2590 NLOS samples; 720 samples are designated as the validation set, which consists of 350 LOS samples and 370 NLOS samples; 1440 samples are designated as the test set, which consists of 700 LOS samples and 740 NLOS samples.

Table 3, Table 4 and Table 5 present the performance of various methods in the underground parking garage (i.e., Scenario A), corridor (i.e., Scenario B), and lobby (i.e., Scenario C), respectively. Our approach achieves a higher NLOS identification accuracy in all the scenarios. In the underground parking garage environment, compared to CNN-MLP-Attention, CDM improves accuracy, precision, and F1-score by 0.14%, 1.52%, and 0.1%, respectively. In the corridor environment, compared to CNN-MLP-Attention, CDM improves accuracy, precision, and F1-score by 0.5%, 0.99%, and 0.49%, respectively. In the lobby environment, compared to CNN-MLP-Attention, CDM improves accuracy, precision and F1-score by 0.32%, 2.03%, and 0.2%, respectively. The higher accuracy achieved by the proposed CDM method indicates that incorporating DAE optimizes feature extraction, thereby improving the overall classification accuracy.

#### 4.2.2. Cross-Scenario NLOS Recognition Performance

We selected the Scenario A as the source domain. The Scenario B and Scenario C were chosen as the target domain. All target samples formed the test set. We directly applied the model trained on Scenario A to unseen environments (i.e., Scenario B and Scenario C) without retraining, yielding the results shown in Table 6. Table 6 compares the NLOS identification accuracy of various models in cross-scenario tests. The results demonstrate that the CDM method achieves the highest recognition accuracy. The model trained in Scenario A is directly applied to the Scenario B and Scenario C, and the NLOS recognition accuracy reaches 75.89% and 72.75%, respectively. Results demonstrate the effectiveness of the model developed in this paper.

To enhance the cross-domain recognition capability of the model, we integrate CDM into the DANN framework, yielding CDMD algorithm. To verify the validity of the developed approach, we compared the proposed method with EDANN-SOR [22] and CNN-BiLSTM+TL [10]. For the CDMD approach, the network parameter settings are shown in Table 2. Other parameters can be found in Table 1. For CNN-BiLSTM+TL [10], we used 0.5% of the target-domain samples to retrain the neural networks. All these methods were evaluated using 20% of the target-domain samples as the test set. Table 7 and Table 8 summarize the identification performance of various methods in the cross-domain transfer scenarios. In cross-domain NLOS recognition from A to B, the proposed CDMD method outperformed CNN-BiLSTM+TL and EDANN-SOR approaches, achieving 3.93% and 4.36% improvement in accuracy, respectively. In the cross-domain NLOS recognition from A to C, the proposed method CDMD outperformed CNN-BiLSTM+TL and EDANN-SOR approaches, achieving 0.22% and 5.42% improvement in accuracy, respectively. In the cross-domain transfer scenario, the proposed algorithm exhibits superior NLOS identification performance.

#### 4.2.3. Impact of Target-Domain Sample Size Variation on the Proposed NLOS Identification Method

To investigate the impact of varying proportions of target-domain samples in the training set on NLOS recognition performance, we conducted corresponding experiments on cross-environment transfer scenarios. We used real collected samples for testing and presented the NLOS recognition accuracies of the proposed method. We split the source-domain data into three subsets: 20% as the test set, 10% as the validation set, and 70% as the training set. The target-domain dataset was partitioned into two independent subsets: 80% as the supplementary training pool, from which a certain proportion of samples was selectively sampled to co-train the proposed model with the source-domain training set, and the remaining 20% as the exclusive target-domain test set (untrained and only used for final performance evaluation). For the cross-environment transfer scenarios of Environment A to B and Environment A to C, the proposed model was trained on 70% of the source-domain samples plus the selectively sampled target-domain samples from the aforementioned 80% supplementary training pool. During model training, the CDMD recognition performance was evaluated on the source-domain validation subset.

The NLOS identification accuracies for the the transition scenario were shown in Table 9. Experimental results demonstrate that when transferring from Scenario A to Scenario B, the developed algorithm achieves an NLOS identification accuracy of 92.50% on the target domain, with only 5% of the target-domain training set samples involved in model training. Similarly, for the transfer from Scenario A to Scenario C, the proposed algorithm reaches 81.76% identification accuracy on the target domain by utilizing merely 5% of the target-domain training set samples for model training. The proposed model can attain an identification accuracy exceeding 80% in cross-environment transfer with merely 5% of the target-domain training samples. The results indicate that merely a small number of target-domain samples can enable the model to achieve high-accuracy cross-domain transfer.

## 5. Conclusions

The identification of LOS and NLOS signals is crucial for UWB communication systems and positioning systems. To address the issues of inadequate accuracy and time-intensive processing in UWB LOS/NLOS signal identification under unmeasured environments, we proposed a novel CDMD approach. In addition, we perform experimental validation on measured datasets collected from three different environments (i.e., underground parking garage, corridor, and lobby). Experiments show that CNN-Attention, MLP-Attention, and CNN-MLP-Attention demonstrate significant performance degradation in cross-domain transfer NLOS recognition tasks. In the same training and testing environment, our proposed method of CDM achieves higher recognition accuracy compared to other approaches. For cross-domain NLOS recognition tasks from A to B and A to C, the proposed method attains accuracies of 77.00% and 72.84%, respectively. Experimental results indicate that the CDMD has higher NLOS identification accuracy than EDANN-SOR and CNN-BiLSTM+TL in the cross-domain transfer NLOS recognition tasks. In addition, when merely 5% of target-domain samples are employed for training, the identification accuracy can reach above 80%. Our work achieved improved cross-domain recognition performance at the cost of a moderate increase in computational complexity, which is mainly attributed to the domain-adversarial feature extractor introduced in the model architecture. The proposed method is thus well-adapted to application scenarios where recognition accuracy and cross-domain robustness are deemed more critical than real-time inference speed. In future research, we will concentrate on the development and optimization of advanced NLOS error compensation methods to address the critical issue of degraded ranging and positioning accuracy caused by NLOS propagation.

## Figures and Tables

**Table 1:** Parameters information of the proposed method.

Parameter	Parameter Selection
Batch size	32
Epochs	250
Optimizer	Adam
Learning rate	CNN: 0.0004, MLP: 0.0065, attention mechanism: 0.0004, Label predictor: 0.0035, Domain predictor: 0.0027
Loss function	Cross entropy loss

**Table 2:** The network structure of the proposed method.

Module	Name	Layer Structure	Output Shape
Input	-	CIR signal input	1×1016
Additional input	-	Static handcrafted features input	1×11
CIR feature extractor	Conv1	Conv1d(1, 32)	32×1016
	MaxPooling1	kernel size = 2, stride = 2	32×508
	Conv2	Conv1d(32, 64)	64×508
	Flatten	-	1×32,512
Handcrafted feature extractor	Encoder: Linear	Linear(11, 16)	1×16
	Decoder: Linear	Linear(16, 11)	1×11
	MLP: FC1	Linear(16, 32)	1×32
	MLP: FC2	Linear(32, 16)	1×16
Feature fusion	CIR feature transformation	Linear(32,512, 64)	1×64
	Static feature transformation	Linear(16, 64)	1×64
	Attention weight calculation	Concat, Linear(128, 64) + Tanh + Dropout, Linear(64, 2) + Softmax	1×2
	Weighted concatenation	Concatenation	1×128
Label predictor	FC1	Linear(128, 64)	1×64
	FC2	Linear(64, 32)	1×32
	FC3	Linear(32, 2)	1×2
Domain classifier	FC1	Linear(128, 64)	1×64
	FC2	Linear(64, 32)	1×32
	FC3	Linear(32, 2)	1×2

**Table 3:** Performance comparison of various methods in Scenario A.

Method	Accuracy (%)	Precision (%)	Recall (%)	F1-Score (%)
CNN-Attention	97.50	96.81	98.38	97.59
MLP-Attention	92.36	90.28	95.41	92.77
CNN-MLP-Attention	97.71	96.45	99.19	97.80
CNN-DAE-MLP-Attention	97.85	97.97	97.84	97.90

**Table 4:** Performance comparison of various methods in Scenario B.

Method	Accuracy (%)	Precision (%)	Recall (%)	F1-Score (%)
CNN-Attention	98.50	98.71	98.29	98.50
MLP-Attention	95.86	98.49	93.14	95.74
CNN-MLP-Attention	98.71	98.58	98.86	98.72
CNN-DAE-MLP-Attention	99.21	99.57	98.86	99.21

**Table 5:** Performance comparison of various methods in Scenario C.

Method	Accuracy (%)	Precision (%)	Recall (%)	F1-Score (%)
CNN-Attention	92.77	92.94	92.53	92.73
MLP-Attention	82.75	85.37	78.90	82.01
CNN-MLP-Attention	92.94	91.94	94.07	92.99
CNN-DAE-MLP-Attention	93.26	93.97	92.42	93.19

**Table 6:** Accuracy comparison of various methods in the cross-domain transfer (%).

Method	A → B	A → C
CNN-Attention	73.69	68.06
MLP-Attention	67.86	50.32
CNN-MLP-Attention	73.99	65.91
CNN-DAE-MLP-Attention	75.89	72.75

**Table 7:** Performance comparison of various methods in the cross-domain transfer (A → B).

Method	Accuracy (%)	Precision (%)	Recall (%)	F1-Score (%)
EDANN-SOR [22]	72.64	85.62	54.43	66.55
CNN-BiLSTM+TL [10]	73.07	81.98	59.14	68.71
CDMD (proposed method)	77.00	78.72	74.00	76.29

**Table 8:** Performance comparison of various methods in the cross-domain transfer (A → C).

Method	Accuracy (%)	Precision (%)	Recall (%)	F1-Score (%)
EDANN-SOR [22]	67.42	72.40	55.93	63.11
CNN-BiLSTM+TL [10]	72.62	70.83	76.59	73.60
CDMD (proposed method)	72.84	67.14	89.12	76.58

**Table 9:** Impact of target-domain sample proportion variation on NLOS recognition accuracy (%).

Identification Accuracy	Percentage of Collected Target-Domain Samples				
	**1%**	**5%**	**10%**	**20%**	**80%**
A → B	82.36	92.50	94.71	96.64	97.71
A → C	74.21	81.76	85.87	88.77	92.39

## Data Availability

Data is contained within the article.
